# Breast Volume Is a Predictor of Higher Heart Dose in Whole-Breast Supine Free-Breathing Volumetric-Modulated Arc Therapy Planning

**DOI:** 10.3390/curroncol30120768

**Published:** 2023-12-18

**Authors:** Rita Alaimo, Edy Ippolito, Rita Falconi, Francesca Perrone Congedi, Cecilia Sciommari, Sonia Silipigni, Roberto Pellegrini, Alessia Carnevale, Carlo Greco, Michele Fiore, Rolando M. D’Angelillo, Sara Ramella

**Affiliations:** 1 Radiation Oncology, Fondazione Policlinico Universitario Campus Bio-Medico, Via Alvaro del Portillo, 200, 00128 Rome, Italy; r.alaimo@policlinicocampus.it (R.A.); f.perronecongedi@policlinicocampus.it (F.P.C.); c.sciommari@policlinicocampus.it (C.S.); s.silipigni@policlinicocampus.it (S.S.); a.carnevale@policlinicocampus.it (A.C.); c.greco@policlinicocampus.it (C.G.); m.fiore@policlinicocampus.it (M.F.); s.ramella@policlinicocampus.it (S.R.); 2 Department of Radiation Oncology (Medicine and Surgery), Università Campus Bio-Medico di Roma, 00128 Rome, Italy; 3 Medical Physics Unit, S. Filippo Neri Hospital, ASL Roma 1, 00135 Rome, Italy; rita.falconi@aslroma1.it; 4 Elekta AB, 113 57 Stockholm, Sweden; roberto.pellegrini@elekta.com; 5 Radiation Oncology, Tor Vergata University, 00133 Rome, Italy; profrmdangelillo@gmail.com

**Keywords:** breast cancer, VMAT, heart dose

## Abstract

In breast cancer volumetric-modulated arc therapy (VMAT) planning, the rotation of the gantry around the target implies a greater dose spreading to the whole heart, compared to tangential-field standard treatment. A consecutive cohort of 121 breast cancer patients treated with the VMAT technique was investigated. The correlation of breast volume, heart volume and lung volume with mean heart dose (mHD) and mean and maximum LAD dose (mLAD dose, MLAD dose) was tested, and a subsequent a linear regression analysis was carried out. VMAT treatment plans from 56 left breast cancer and 65 right breast cancer patients were analyzed. For right-sided patients, breast volume was significantly correlated with mHD, mLAD and MLAD dose, while for left-sided patients, breast volume was significantly correlated with mHD and mLAD, while heart volume and lung volume were correlated with mHD, mLAD and MLAD dose. Breast volume was the only predictor of increased heart and LAD dose (*p* ≤ 0.001) for right-sided patients. In left-sided patients, heart and lung were also predictors of increased mHD (*p* = 0.005, *p* ≤ 0.001) and mean LAD dose (*p* = 0.009, *p* ≤ 0.001). In this study, we observed an increase in heart and LAD doses in larger-breasted patients treated with VMAT planning. In right-sided patients, breast volume was shown to be the only predictor of increased heart dose and LAD dose.

## 1. Introduction

Radiotherapy (RT) is an established part of the multidisciplinary treatment of breast cancer. After breast-conserving surgery, adjuvant RT reduces the risk of loco-regional recurrence and is widely used as the standard of care [1]. Several fractionation schemes and treatment modalities have been applied and investigated. However, adjuvant treatments may have some detrimental late effects on nearby healthy organs.

In particular, there are data regarding patients with left-side breast cancer treated with older techniques, where radiotherapy increases the risk of heart disease [2,3]. A meta-analysis recorded a link between cardiac deaths following breast radiotherapy and the volume of the heart receiving 5 Gy [4].

Several predictors of heart exposure during RT for breast cancer have been identified, including clinical, anatomical and planning factors [5,6,7,8,9,10]. Among these, larger PTV, requiring a longer distance between the medial and lateral entry points and a larger portion of the heart in the radiation field, was found to worsen heart dosimetry [9]. However, most studies included only patients treated with three-dimensional conformal RT (3D-CRT).

Since the introduction of volumetric-modulated arc therapy (VMAT) after the publication of the seminal work of Otto [11] and the subsequent implementation of optimization algorithms in treatment planning systems, VMAT has been applied to almost all type of cancers. When this technique is used for breast cancer postoperative treatment, the rotation of the gantry around the target implies a greater dose spreading to the whole heart compared to tangential-field treatment [12]. This can be even greater in cases of treatment of large-breasted patients. For this reason, we wanted to evaluate how breast volume affects heart dose in supine free-breathing VMAT radiotherapy planning for breast cancer patients.

## 2. Materials and Methods

### 2.1. Patient Selection

We retrospectively investigated a consecutive cohort of 56 left breast cancer and 65 right breast cancer patients treated with the VMAT technique. All patients were consecutively treated at the University Campus Bio-Medico of Rome, Italy, from June 2022 to December 2022. Patients enrolled signed a consent form for data collection according to the study design requirements and Fondazione Policlinico Universitario Campus Bio-Medico ethical committee.

### 2.2. Simulation and Target Definition

All patients were positioned supine on a C–Qual M™ Breastboard (angle 10–12 degrees) with both arms lifted up above the head. The planning CT was performed on a 16-slice Computed Tomography (CT) scanner (Somatom Sensation CT-scanner, Siemens Medical Systems, Erlangen, Germany) with a slice thickness of 3 mm. Clinical target volume (CTV), planning target volume (PTV) and organs at risk (OARs) were delineated according to ESTRO guidelines for breast cancer [13]. For heart contouring, the atlas by Feng et al. was used [14]. The breast CTV included the breast volume, after a reduction of 5 mm from the surface edge, not taking into account the major pectoral muscle, the lung and the ribs. The breast PTV was defined with a 5 mm margin around the CTV.

### 2.3. Treatment Planning

For all patients, we employed the VMAT technique. The dose calculation was performed using the Montecarlo (MC) Algorithm provided by the Monaco 5.51.10 Treatment Planning System (Elekta A.B., Stockholm, Sweden). A grid calculation size of 3 mm was used with 1% statistical MC variance. The plan design consisted of two small tangential arcs (each partial arc, geometrically resembling the 3D-CRT tangential beams, consisted of four arcs spanning 40–60 degrees amplitude each: the first in the clockwise direction follow by another in the anti-clockwise direction, the third again covering 40–60 degrees amplitude in the clockwise direction and the last coming back to the start point) with 6 MV photons, aiming to conform the prescribed dose to the breast target, reducing cardiac and lung doses. Since breast cancer extends towards the patient’s surface, the target volume could move outside the treatment field. In order to take into account this issue, an Auto Flash margin (value from 1.5 cm up to 2.5 cm) was used, leading the multileaf collimator (MLC) leaves opening outside of the body contour. The prescribed dose was 40.05 Gy (2.67 Gy/d). The optimization objectives were as follows: 95% of prescription dose to 95% of the PTV volume; 105% of prescription dose to less than 5% of PTV volume. For the OARs, the following constraints were used: heart mean dose < 5 Gy (optimal < 3.5 Gy), left descending artery (LAD) Dmax < 20 Gy (optimal < 15 Gy), LAD Dmean < 8 Gy, volume of lung receiving 5 Gy- V5 < 60%, contralateral breast mean dose < 3 Gy. Treatments were delivered with two energy-matched ELEKTA VERSAHD Linac devices (Elekta, Crawley, UK). Patient positioning was performed with the AlignRT Advance (Vision RT, Ltd., London, UK) SGRT system, which provides a real-time motion monitoring of the surface. The real-time surface was compared with the CT planning surface that was set as reference. An additional position verification was performed with a daily CBTC in order to make sure the internal OARs’ location was correct. All patients were treated with free breathing.

### 2.4. Statistical Analysis

The treatment plans were evaluated using the ProKnow data analysis platform (Elekta). The dose–volume parameters for each OAR and anatomic volumes such as breast volume, heart volume and lung volume were recorded for each patient. Comparisons by groups according to breast volume (cut-off = mean breast volume, 892 cc) was performed using the paired *t*-test. The correlation of breast volume, heart volume and lung volume with mHD, mean dose and maximum dose of LAD (mLAD, MLAD, respectively) was tested using Pearson’s correlation coefficient “r”. The variance inflation factor (VIF) was used to judge whether there was collinearity among variables. The volumes that significantly correlated with mHD, mLAD and MLAD dose were examined using a linear regression model. A linear regression model was created using the variable with a significant value of the correlation coefficient. Linear regression requires that residuals conform to normal analysis and are independent of each other, so residuals of each regression model were calculated, and histograms were used to explore whether the residuals conformed to normal distribution. The Durbin–Watson test was used to test whether the residuals in the linear regression model were independent of each other. Variables with a *p* < 0.01 in the univariate regression model were evaluated in the multivariate regression model. Statistical analysis was performed using IBM SPSS (Statistical Package for the Social Sciences) v.26 (APA, MLA, Chicago, IL, USA).

## 3. Results

### 3.1. Patients’ Characteristics

In this study, 121 patients affected by breast cancer receiving adjuvant whole-breast RT were taken into account. The mean age of the whole patient population was 65.2 years (SD 11.12 years). Of these patients, 56 patients were treated for left breast cancer, and 65 patients were treated for right breast cancer. The mean breast volume for the whole population was 892.00 cc (SD 389.77), 892.64 cc (SD 435.56) in left-sided patients and 864.09 cc (SD = 348.45) in right-sided patients, respectively. Dosimetric parameters (mean value, SD) for right-sided and left-sided breast cancer patients are summarized in Table 1.

**Figure 1:**
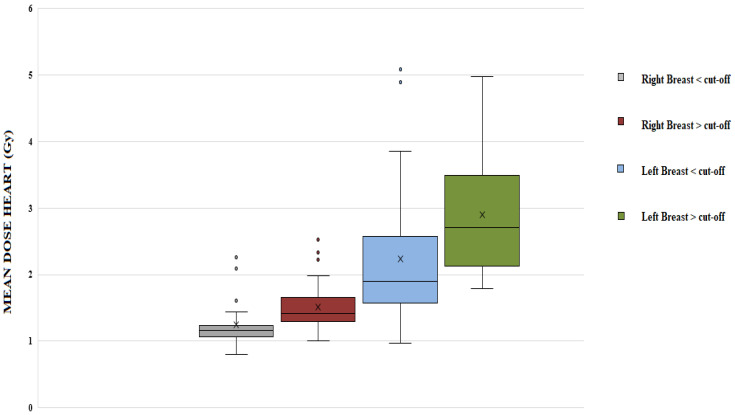
Dosimetric parameters (mean dose heart, max dose LAD, mean Dose LAD) for right-sided and left-sided (cut-off = mean breast volume, 892 cc).

mHD was 1.4 Gy (SD 0.40) for right-sided patients and 2.5 Gy (SD 1.0) for left-sided patients. In right-sided patients, mHD (*p* = 0.03), mLAD (*p* = 0.015) and MLAD (*p* = 0.07) were significantly higher in patients with larger breast volume (cut-off value = 892 cc). Their values increased by 25%, 18% and 13%, respectively. In left-sided patients, mHD was increased by 30% (*p* = 0.01) in patients with a larger breast volume (see Figure 1). No significant differences were observed in mLAD and MLAD.

Breast volume, heart volume and lung volume did not exceed the maximum acceptance VIF level and therefore were included in the following statistical analysis.

### 3.2. Correlation Analysis

The results of correlation analysis with mHD, mLAD and MLAD dose are shown in Table 2. For right-sided patients, breast volume was significantly correlated with mHD, mLAD and MLAD dose, while lung volume was significantly inversely correlated to mHD only. For left-sided patients, breast volume was significantly correlated to mHD and mLAD, while heart volume and lung volume were correlated (lung inversely) with mHD, mLAD and MLAD dose.

### 3.3. Regression Analysis

The results for regression analysis are summarized in Table 3.

#### 3.3.1. Right-Sided Patients

**Figure 2:**
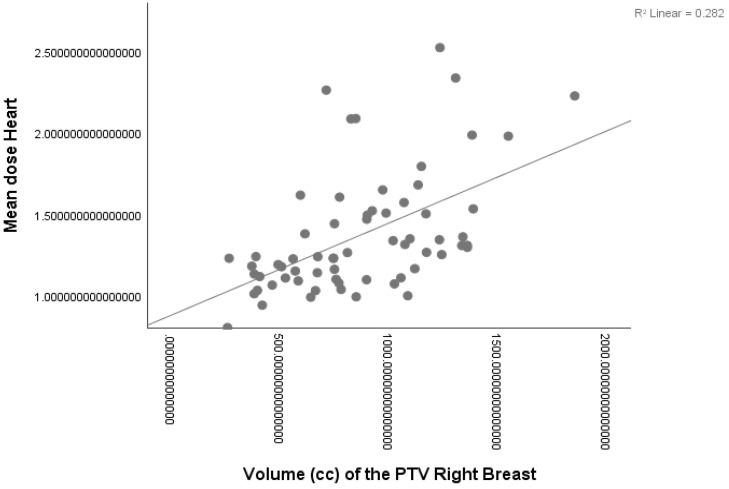
Linear regression fitting model of mHD in right-sided patients.

Larger breast volumes were associated with increased mHD, mLAD and MLAD. Lung volume did not show a strong relationship with the heart dose variable (*p* = 0.024) and therefore a multivariate analysis was not performed. The following formulas specify the univariate linear regression fitting model for mHD (a, see Figure 2), mLAD (b) and MLAD (c):

(a) mHD (Gy) =0.881 + (0.531 × breast volume)
(b) mLAD (Gy) = 0.860 + (0.443 × breast volume)
(c) MLAD (Gy) =1.100 + (0.323 × breast volume)

The mean (SD) of absolute residuals was 0.0 (0.2) Gy for (a), 0.0 (0.3) Gy for (b) and 0.0 (0.5) Gy for (c). The value of the Durbin–Watson test was 2.0 for (a), 1.92 for (b) and 2.06 for (c), showing the independence of residuals.

#### 3.3.2. Left-Sided Patients

The results for regression analysis are summarized in Table 3. Breast volume was only shown to be highly related to mHD in univariate analysis, not in multivariate analysis. Heart volume and lung volume were shown to be significantly related to mHD and mLAD dose in both univariate and multivariate analysis. Lung volume was only the best predictor of increased MLAD dose. The following specify the multiple linear regression fitting model of mHD (a) and mLAD dose (b):

(a) mHD (Gy) = 1.705 + (0.208 × breast volume) + (0.312 × heart volume) − (0.411 × lung volume)
(b) mLAD dose (Gy) = 6.528 + (0.281 × heart volume) − (0.565 × lung volume)

The mean (SD) of absolute residuals was 0.0 (0.7) Gy for (a) and 0.0 (1.6) Gy for (b). The value of the Durbin–Watson test was 2.0 for (a) and 2.1 for (b), showing independence of residuals.

The following equation specifies the linear regression fitting model of MLAD dose:

(a) MLAD dose (Gy) = 22.812 − (0.639 × lung volume)

The mean (SD) of absolute residuals was 0.0 (3.0) Gy. The value of the Durbin–Watson test was 2.2, showing the independence of residuals.

## 4. Discussion

In this study, we analyzed heart radiation exposure using VMAT radiotherapy planning with regard to breast volume in a cohort of 121 breast cancer patients treated with whole-breast RT only (WBRT). In all patients, predefined mandatory heart constraints were respected in both left- and right-sided patients. However, recent guidelines recommend more stringent heart constraints [15] for whole-breast radiotherapy. Considering these latter constraints, we would have exceeded the recommended dose in 9% of right-sided and 45% of left-sided patients.

In the whole patient population, larger-breasted patients showed increased mHD by 25% and 30% in right and left patients, respectively. However, the contribution of breast volume in left-sided patients to heart and LAD doses was shown to be less relevant compared to the lung and heart volume. On the contrary, in right-sided patients, breast volume seems to be the main predictor of increased heart and LAD doses.

Several studies have investigated anatomical and clinical predictors of heart exposure in 3D breast radiotherapy and the relative benefit from DIBH in left breast cancer patients. Among anatomical factors, smaller lung volumes [5,6], as well as the cardiac contact distance measured on different planes and larger PTV [7,8,9], were considered predictors of increased heart exposure. Among clinical factors, higher BMI and vital capacity measured by spirometry were predictive of higher heart dose [10].

The introduction of intensity-modulated radiotherapy (IMRT) compared with the use of 3D-CRT was associated with greater mHD [16]. However, few studies investigated factors influencing heart exposure in patients treated with intensity-modulated techniques. Kang et al. evaluated cardiac junction and pulmonary junction (anatomic variable derived from 3D plans) to predict the benefit from the use of VMAT in patients with left-sided breast cancer [17]. 

Different heart-sparing techniques have been investigated [18]. Among them, several studies and consistent literature data support the use of the deep inspiration breath hold (DIBH) technique, which can reduce radiation-induced cardiac toxicity by increasing the distance between the breast and the heart [19,20,21,22].

Despite the widespread usage of DIBH for left-breast RT, few studies have investigated its role in right-breast cancer [23,24,25]. Some studies suggested that DIBH for right-sided breast cancer should be adopted to reduce ipsilateral lung and liver dose in loco-regional radiation therapy [26,27]. A significant benefit was detected in reducing the maximum dose to the heart and the right coronary artery in cases of regional nodal irradiation [23,24]. Interestingly, in a study evaluating the dosimetric benefit of DIBH for locoregional irradiation of R-BC with VMAT, Loap et al. concluded that adding DIBH to VMAT is not justified for all patient candidates for right-breast and regional nodal irradiation. Therefore, specific patient subpopulations who could benefit from additional DIBH combination with locoregional VMAT needed to be identified [25].

Also, prone radiotherapy has been investigated in both left- and right-sided patients in terms of dosimetric benefit over supine radiotherapy [28,29,30]. Median higher heart doses were observed in prone radiotherapy both in left-sided patients when compared to DIBH supine radiotherapy (mean dose 3.4 vs. 1.9 Gy) and in right-sided patients when compared with supine radiotherapy (mean dose 1.9 vs. 1.3 Gy) [29,30]. However, the overall dosimetric benefit, taking into account not only heart dose but also lung, PTV and extra-target dose favored prone positioning in 61% of left-sided patients and in 81.5% of right-sided patients, and in both cases, breast volume predicted the benefit from prone positioning [29,30].

This study has some limitations. First, it was retrospective and therefore is subject to selection biases. Second, there is a lack of comparison with alternative techniques, such as DIBH or alternative positioning (prone radiotherapy), to verify if these techniques would obviate the higher heart dose in larger-breasted patients. This would be of great interest and should be investigated in future analyses.

## 5. Conclusions

In conclusion, in this study, we observed an increase in heart and LAD doses in larger-breasted patients undergoing breast-only radiotherapy. In right-sided patients, breast volume was shown to be the only predictor of increased heart dose and LAD dose. Even if the absolute increase in doses was limited, given the low doses of heart dosimetry in these patients, the role of breast volume is certain and should also be further evaluated in patients undergoing regional nodal irradiation when VMAT planning is used. This is of particular relevance as patients are routinely treated with free breathing.

## Figures and Tables

**Table 1:** Dosimetric parameters (mean value, SD) for right-sided and left-sided breast cancer patients.

Variables	Left-Sided Patients				Right-Sided Patients			
N° Patients	56				65			
	Minimum	Maximum	Mean	SD	Minimum	Maximum	Mean	SD
Breast Volume (cc)	233.48	2189.57	892.64	435.56	256.79	1852.58	864.09	348.45
Heart Volume (cc)	436.78	1029.84	643.77	118.20	419.53	1359.37	637.85	150.53
Ipsilateral Lung Volume (cc)	668.87	3076.97	1203.13	363.65	828.87	2291.82	1502.13	336.13
mHD	0.96	5.08	2.55	0.10	0.80	2.52	1.37	0.37
MLAD	3.78	20.15	14.29	4.03	0.97	4.53	1.59	0.62
mLAD	1.81	11.17	5.77	2.24	0.72	2.43	1.24	0.35

Abbreviations: mHD = mean heart dose; mLAD = mean LAD dose; MLAD = maximum LAD dose.

**Table 2:** Correlation analysis between CT volumes (breast, lung, heart) and heart dosimetry in right- and left-sided breast patients.

Side	Volume	mHD (r, *p*)	mLAD (r, *p*)	MLAD (r, *p*)
Right	Breast	0.531, <0.001 *	0.443, <0.001 *	0.323, <0.001 *
	Heart	0.243, 0.051	0.156, 0.215	0.125, 0.323
	Ipsilateral Lung	−0.280, 0.024 *	−0.125, 0.320	−0.130, 0.301
Left	Breast	0.392, 0.003 *	0.327, 0.014 *	0.180, 0.183
	Heart	0.378, 0.004 *	0.345, 0.090	0.332, 0.012 *
	Ipsilateral Lung	−0.524, <0.001 *	−0.597, <0.001 *	−0.639, <0.001 *

Abbreviations: r: Pearson’s correlation coefficient; *p*: *p* value, * statistically significant, LAD = left descending artery; mHD = mean heart dose; mLAD = mean LAD dose; MLAD = maximum LAD dose.

**Table 3:** Linear regression coefficients for prediction of mHD, mean LAD dose and maximum LAD dose (univariate and multivariate).

	Univariate				Multivariate		
				
**Side RIGHT**	**mHD**	**R2**	**F**	***p***	**R2**	**F**	***p***
	Breast Volume	0.282	24.790	<0.001 *			
	Lung Volume	0.085	5.359	0.024			
	**mLAD**						
	Breast Volume	0.196	15.395	<0.001 *			
	**MLAD**						
	Breast Volume	0.104	7.331	0.009 *			
				
**Side LEFT**	**mHD**	**R2**	**F**	***p***	**R2**	**F**	***p***
	Breast Volume	0.154	9.800	0.003 *	0.483	16.165	0.349
	Heart Volume	0.143	9.024	0.004 *			0.005
	Lung Volume	0.274	20.429	<0.001 *			<0.001
	**mLAD**						
	Breast Volume	0.107	6.462	0.014			
	Heart Volume	0.119	7.301	0.009 *	0.434	20.350	0.009
	Lung Volume	0.356	29.893	<0.001 *			<0.001
	**MLAD**						
	Heart Volume	0.110	6.706	0.012			
	Lung Volume	0.408	37.213	<0.001 *			

Abbreviations: mHD = mean heart dose; LAD = left descending artery; mLAD = mean LAD dose; MLAD = maximum LAD dose. * statistically significant.

## Data Availability

The data that support the findings of this study are available from the corresponding author (RA) upon reasonable request.
